# Waltonitone inhibits proliferation of hepatoma cells and tumorigenesis via FXR-miR-22-CCNA2 signaling pathway

**DOI:** 10.18632/oncotarget.12614

**Published:** 2016-10-12

**Authors:** Fan Yang, Junting Gong, Guangyun Wang, Peng Chen, Li Yang, Zhengtao Wang

**Affiliations:** ^1^ The MOE Key Laboratory for Standardization of Chinese Medicines and the SHTCM Key Laboratory for New Resources and Quality Evaluation of Chinese Medicines, Institute of Chinese Materia Medica, Shanghai University of Traditional Chinese Medicine, Shanghai 201203, China; ^2^ First Affiliated Hospital, Guangzhou University of Chinese Medicine, Guangzhou 510006, China; ^3^ Center for Chinese Medical Therapy and Systems Biology, Shanghai University of Traditional Chinese Medicine, Shanghai 201203, China

**Keywords:** WA, farnesoid X receptor, microRNA-22, cyclin A, HCC

## Abstract

Waltonitone (WA), an ursane-type pentacyclic triterpene extracted from *Gentiana waltonii* Burkill, was recently appeared to exert anti-tumor effect. However, the biological underpinnings underlying the role of WA in hepatocellular carcinoma (HCC) cells have not been completely elucidated. Our previous report indicated that the FXR-regulated miR-22-CCNA2 pathway contributed to the progression and development of HCC. Besides, a wide spectrum of microRNAs (miRNAs) could be up- or down-regulated upon WA treatment, including miR-22. Hence, we aimed to determine whether WA inhibited HCC cell proliferation via the FXR-miR-22-CCNA2 axis. In this study, we observed a significant downregulation of FXR and miR-22, along with upregulation of CCNA2 in 80 paired tumors relative to adjacent normal tissues of HCC subjects, which were obtained from the available GEO database in NCBI (GSE22058). Furthermore, we validated the expression patterns of these three targets in another set of HCC samples and found the highly correlation within each other. Additionally, our data demonstrated that WA induced miR-22 and repressed CCNA2 in HCC cells, which contributed to the cell proliferation arrest. In addition, evidence suggested that either miR-22 silencing or FXR knockdown reversed the diminished CCNA2 expression as well as cell proliferation inhibition caused by WA treatment and WA inhibited tumor masses *in vivo* in a subcutaneous xenograft mouse model of HCC. Overall, our data indicated that WA inhibited HCC cell proliferation and tumorigenesis through miR-22-regulated CCNA2 repression, which was at least partially through FXR modulation.

## INTRODUCTION

Triterpenes and their derivatives are widely distributed in plants and herbs [1], most of which were well known to exert anti-tumor properties [2–8]. Our previous research identified waltonitone (WA), an ursane-type pentacyclic triterpene, as an anticancer reagent out of 40 chemical constituents derived from *Gentiana waltonii* Burkill. Notably, increasing evidence indicated that WA inhibited tumor progression by modulating some molecules and signal transduction pathways [9–11]. For instance, WA induced lung cancer cell apoptosis via microRNAs regulation [9], and miR-663-repressed Bcl-2 pathway was the predominant one [10]. Moreover, miR-22, which functioned as a tumor suppressor gene, was activated after WA treatment in lung cancer cells [9]. WA also induced hepatocellular carcinoma (HCC) cell apoptosis through the regulation of Bcl-2 family, as supported by our previous study [11]. However, the biological underpinnings underlying the role of WA in HCC cell death through proliferation remains largely unknown. Thus, in the present study, we sought to explore the new molecular mechanism of WA in HCC through miR-22 modulation, and then provide evidence and rational strategy to further pursue for improving HCC treatment.

Farnesoid X Receptor (FXR) serves as a hepatic protector and was proposed to play a dominant role in tumor progression [12–16]. The downstream targets driven by FXR have been increasingly recognized to exert robust impact on HCC development, including microRNAs. MicroRNAs, which are responsible for the post-transcriptional regulation of target mRNAs, function as effective suppressors in different cancers. Various miRNAs exhibited abnormal expressions in HCC tissues relative to non-tumor liver samples. These miRNAs do not only serve as useful clinical biomarkers but are also potential therapeutic targets for HCC treatment. In our previous study, FXR-induced miR-22 significantly affected HCC cell proliferation through CCNA2 repression [17]. In the present study, we further discovered that the downregulation of FXR and miR-22 were highly associated with the upregulation of CCNA2 in tumor tissues relative to normal ones of HCC specimens. The data were obtained from downloaded GEO database of NCBI (GSE22058) and the expressions of these targets were validated in another set of HCC samples. Thus, we sought to determine whether WA could inhibit HCC cell proliferation via the FXR-miR-22-CCNA2 axis. Evidence presented *in vitro* and *in vivo* suggested that WA inhibited HCC cell proliferation and tumorigenesis through miR-22-repressed CCNA2, which was at least partially through FXR modulation. These results prompted WA as a potential therapy or a complementary and alternative option for further HCC treatment.

## RESULTS

### WA induced HCC cell death in a dose- and time-dependent manner

**Figure 1:**
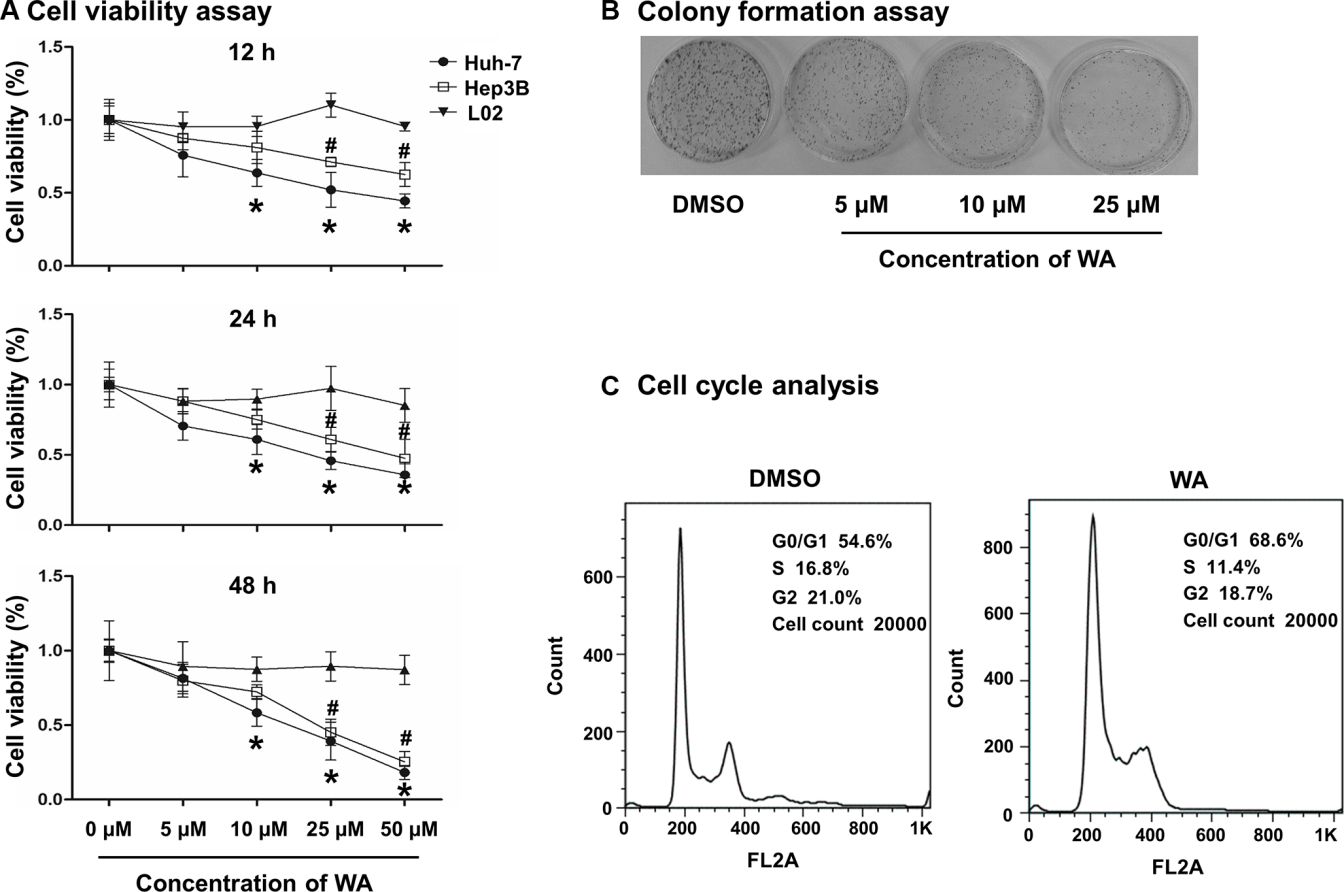
WA induced cell death and cell growth arrest WA at doses of 5, 10, 25 and 50 μM were applied in Huh7, Hep3B and L02 cells for cell viability study (**A**). Different time points (12, 24 and 48 h) were studied after WA treatment (A). WA were treated to Huh7 cells for the colony assay study at concentrations of 5, 10 and 25 μM for 15 days prior to the clear observation of colonies (**B**). Cell cycle analysis was conducted in response to WA treatment at concentration of 25 μM by using flow cytometer (**C**). All data are expressed as mean ± S.D. ^*^p < 0.05 Huh7 cell versus L02 cell; ^#^*p* < 0.05 Hep3B versus L02 cell. ^*^p < 0.05 DMSO versus WA;

To address the inhibitory role of WA in HCC cells, we assessed the cell viability in HCC cells (Huh7 and Hep3B) and normal liver cells (L02) after WA exposure. Different concentrations of WA ranging from 5 μM to 50 μM and a time-course experiment (12 h to 48 h) were applied to the cells. As shown in Figure 1A, WA distinctly induced cancer cell death in a dose- and time-dependent manner. As expected, normal liver cell viability was not altered by WA treatment, indicating that WA is a potential and specific anti-cancer compound. The results demonstrated that WA exerted more cytotoxic sensitivity to cell death in Huh7 cell line. Hence, further experiments were conducted on Huh7 cells.

### WA inhibited HCC cell proliferation

Colony formation assay demonstrated a significant decrease in the number of colony-forming units in response to WA treatment (Figure 1B), showing the inhibitory effect of WA on cell proliferation. Additionally, to further investigate the inhibitory role of WA on cell-cycle regulation, Huh7 cells treated with WA were subjected to flow cytometry. As shown in Figure 1C and Supplementary Figure S1A, WA significantly increased the percentage of Huh7 cells in G0/G1 phase and decreased the percentage of S-phase cells, suggesting that cell proliferation was inhibited by WA.

### Repression of FXR and miR-22, along with upregulation of CCNA2, were observed in tumor tissues relative to adjacent normal ones in HCC patients

**Figure 2:**
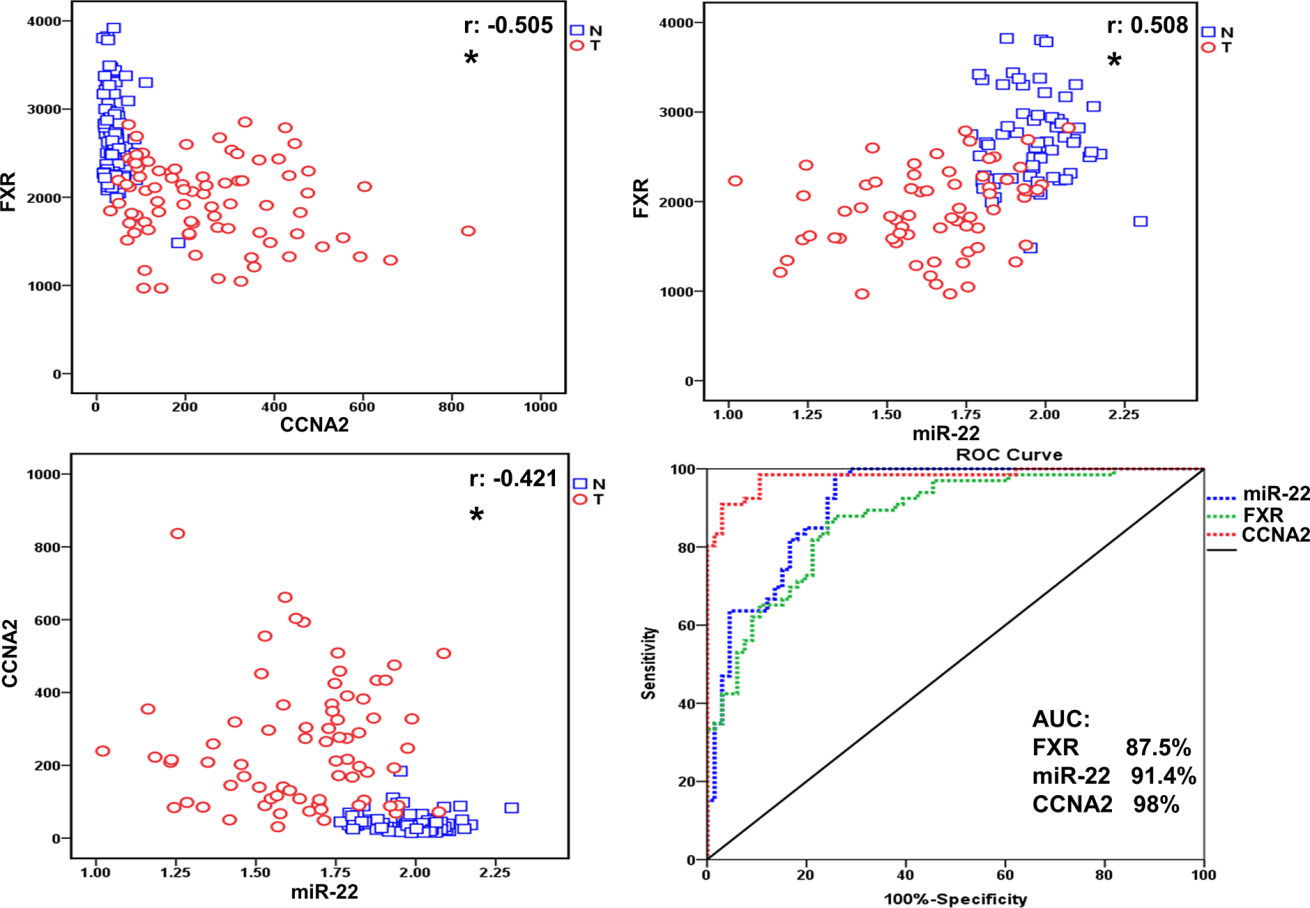
Repression of FXR and miR-22, along with upregulation of CCNA2, were observed in tumor tissues relative to adjacent normal ones in HCC patients The correlation and relative expression were visualized in the plot. The blue open box represents normal tissues and red open circle represents the tumor ones. The correlation between FXR, miR22 and CCNA2 were valued by Pearson correlation coefficient (r, ^*^p < 0.05.). The ROC curve is obtained by plotting sensitivity on the y-axis against specificity on the x-axis.

To identify the underlying mechanism of WA in HCC cell proliferation, we therefore analyzed the relative expressions of FXR, miR-22, and CCNA2 in both tumor and adjacent normal tissues derived from HCC patients (data downloaded from GEO database in NCBI, GSE22058). We showed that CCNA2 was highly expressed along with the pronounced downregulation of FXR and miR-22 in a considerable proportion of the tumor tissues in comparison to noncancerous ones (Figure 2). The expression levels of FXR and miR-22 showed a positive correlation (*r* = 0.508), whereas a negative correlation existed between FXR and CCNA2 (*r* = −0.505). The same correlation was observed between miR-22 and CCNA2 (r = −0.421). The area under the curve (AUC) of FXR (AUC = 87.5%), miR-22 (AUC = 91.4%), and CCNA2 (AUC = 98%) exhibited good sensitivity for tumor prediction (Figure 2). Notably, we also observed the same pattern of FXR, miR-22 and CCNA2 expressions in another set of HCC specimens (Supplementary Figure S2), the samples were obtained from the Translational Pathology Core Laboratory at the University of California, Los Angeles and described in our previous study [17]. Together, the repression of FXR and miR-22, along with the high expression of CCNA2, were apparently observed in HCC tumor tissues, either of which demonstrated high correlation with the other.

### WA induced the expression of miR-22 and repressed the protein level of CCNA2

**Figure 3:**
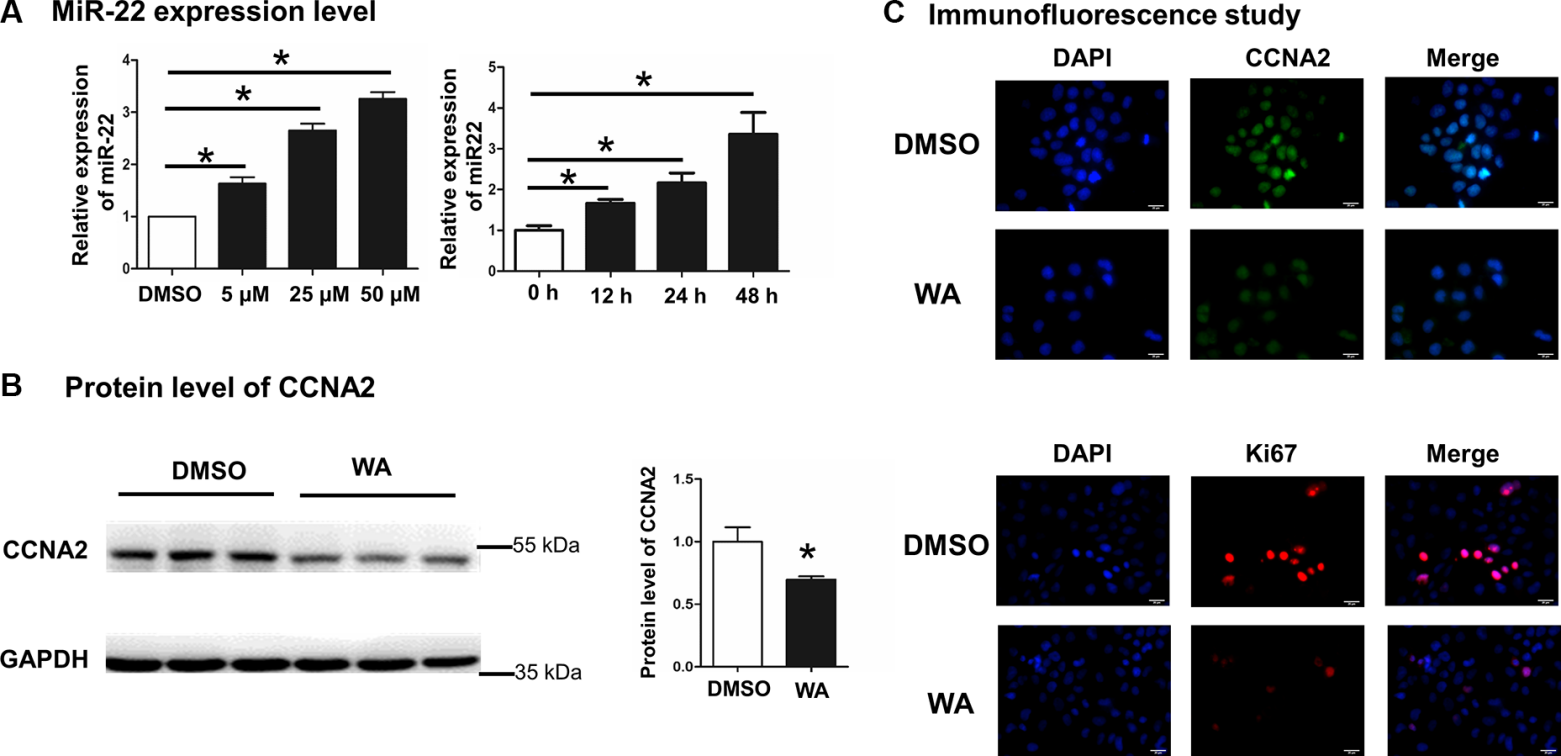
WA treatment induced miR-22 expression and repressed CCNA2 protein level Dose-responsive and time-course experiments were conducted by WA treatment for 48 h and the expression level of miR-22 was analyzed by Real time-PCR (**A**). The protein level of CCNA2 was detected by western blot after WA treatment at dose of 25 μM for 48 h (**B**). Immunofluorescence study was performed by using anti-CCNA2 and anti-ki67 antibodies after WA treatment at dose of 25 μM for 48 h (**C**). Data are presented as mean ± SD. ^*^p < 0.05.

Our previous study demonstrated that FXR transcriptionally regulated miR-22 and repressed CCNA2 expression in HCC cell line. Thus, we thereby investigated whether WA can inhibit cellular proliferation through FXR-miR-22-CCNA2 pathway in Huh7 cell. Our data revealed a dose- and time-dependent induction of miR-22 expression after WA treatment (Figure 3A). Cyclin genes are the dominant regulators in cell-cycle progression. Through western blot analysis, protein level of CCNA2 (one of the important cell-cycle genes) was remarkably inhibited upon WA treatment (Figure 3B). In addition, the immunofluorescence expression of cell-cycle regulator (CCNA2) in association with proliferation factors (Ki-67) were detected using antibodies against CCNA2 or Ki-67. Together with the observation on the diminished expression of CCNA2 and Ki67 after WA exposure (Figure 3C), our data identified that inducing miR-22 and repressing CCNA2 expression by WA were potentially associated with the outcome of cell proliferation inhibition.

### WA inhibited CCNA2 protein level in a miR-22-dependent manner

**Figure 4:**
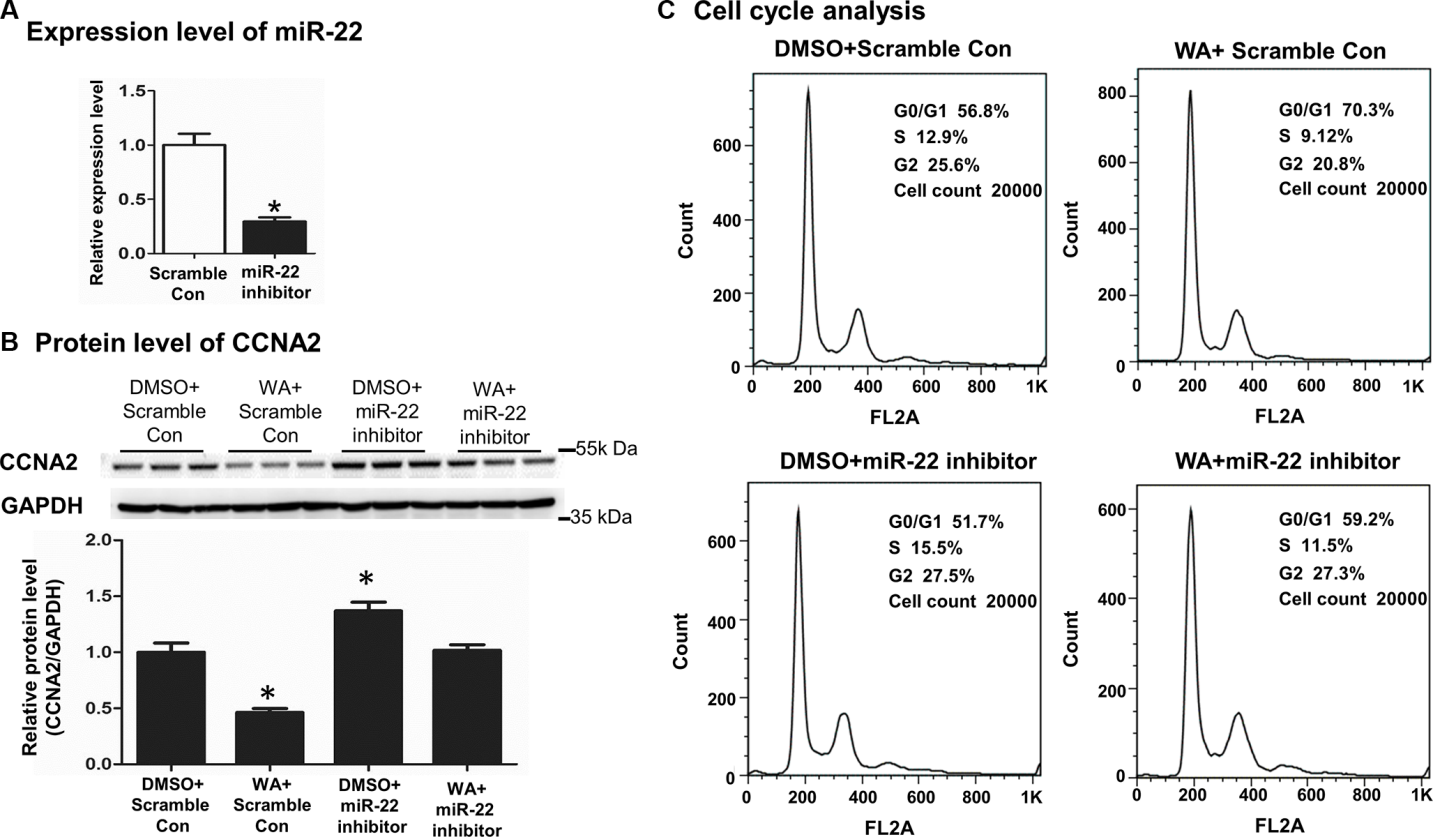
MiR22 silencing reversed the CCNA2 expression and the cell proliferation inhibition caused by WA treatment Scramble control (50 nM) or miR-22 (50 nM) inhibitor were transfected into Huh7 cells. Forty-eight hours later, the expression of miR-22 were analyzed by Real time-PCR (**A**). After transfection with miR-22 inhibitor or scramble control for 6 h, WA (25 μM) or DMSO were treated for 48 h. Protein level of CCNA2 was measured by western blot (**B**) and cell cycle analysis was conducted by using flow cytometer (**C**). Data are presented as mean ± SD. ^*^p < 0.05, versus DMSO+Scramble Con.

To further delineate whether the repression of CCNA2 by WA was miR-22-dependent, we applied the miR-22 inhibitor to elaborate on this point. First of all, we determined the miR-22 level to verify the effectiveness of the inhibitor transfection. Second, quantitative PCR analysis showed that the expression of miR-22 was decreased by 70% after transfection (Figure 4A). Given the loss-of-function of miR-22, we observed that the protein level of CCNA2 remained unchanged upon miR-22 silencing after WA treatment relative to the corresponding untreated ones (Figure 4B), suggesting that the inhibition of CCNA2 by WA was through miR-22 inhibition. Simultaneously and finally, we investigated the different phases of cell-cycle distribution using a flow cytometer. The G0/G1 phase cell population dramatically decreased compared with that of WA treatment alone in the context of miR-22 silencing followed by WA exposure (Figure 4C and Supplementary Figure S1B). Thus, evidence suggested that miR-22 silencing reversed the WA-induced CCNA2 repression and the inhibition of cell proliferation.

### WA inhibited CCNA2 protein level in a FXR-dependent manner

**Figure 5:**
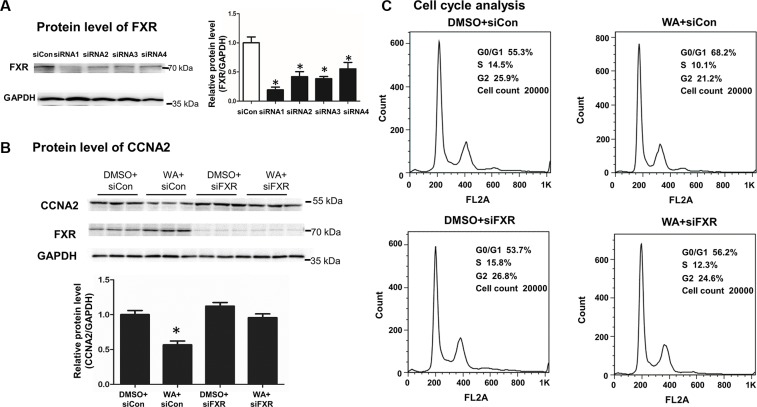
FXR silencing abolished the CCNA2 expression and the cell proliferation inhibition caused by WA treatment The inhibitory efficacy of FXR was analyzed by western blot within different siFXRs (100 nM) (**A**). After transfection with siFXR or negative control for 6 h, WA (25 μM) or DMSO were treated for 48 h. Protein level of CCNA2 was measured by western blot (**B**) and cell cycle analysis was conducted by using flow cytometer (**C**). Data are presented as mean ± SD. ^*^p < 0.05, versus DMSO+siCon.

MiR-22 was transcriptionally regulated by FXR, and WA could repress CCNA2 through miR-22 regulation. Thus, determining whether FXR regulation contributed to the repression of CCNA2 is crucial. FXR knockdown in Huh7 cells was initially obtained with four siRNAs. The first siRNA was selected for further functional research because it was proven to exert the profoundly knockdown effect (Figure 5A). Upon successful FXR inhibition, WA failed to repress the protein level of CCNA2 (Figure 5B) and increase the number of G0/G1 cells (Figure 5C and Supplementary Figure S1C). The above results demonstrated that knockdown of FXR abolished the cell proliferation inhibition and the repression of CCNA2 caused by WA treatment.

### WA inhibited tumor masses *in vivo* in a subcutaneous xenograft mouse model of HCC

**Figure 6:**
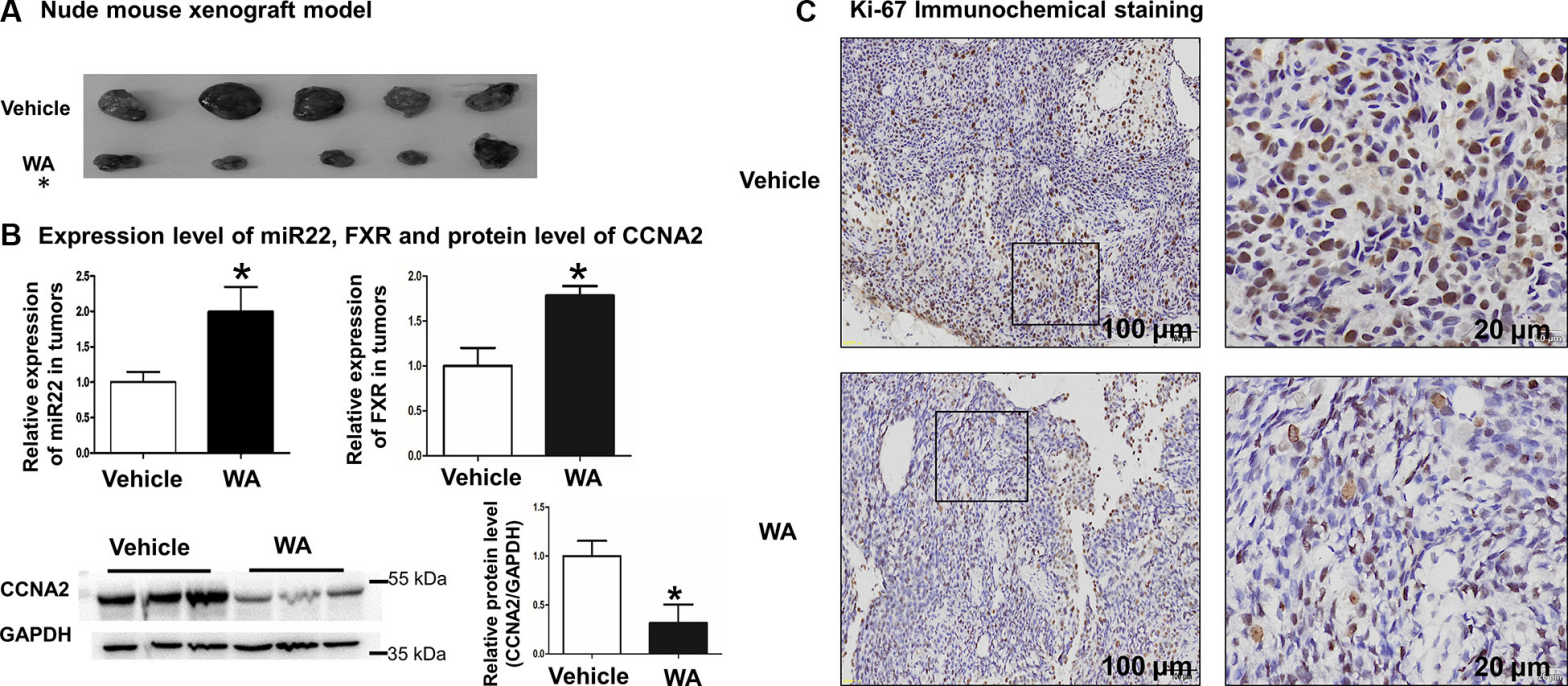
WA inhibited tumor masses *in vivo* in a subcutaneous xenograft mouse model of HCC WA treatment significantly reduced the size of tumors (**A**). MiR22 and FXR expression level along with CCNA protein level were detected in tumors of WA-treated or untreated mice (**B**). Immunohistochemical staining was conducted by using anti-ki67 antibody in tumors of WA-treated or untreated mice (**C**). Data are presented as mean ± SD. ^*^p < 0.05.

Finally, a BALB/c nude mouse xenograft model was applied to assess the effect of WA on tumorigenesis. No differences between the body weights of mice were observed in two groups (Supplementary Figure S3 upper), and WA treatment caused less tumor formation and significantly decreased tumor size compared with the non-treated ones (Figure 6A and Supplementary Figure S3 lower). In addition, the expression of miR-22, FXR, along with the protein level of CCNA2, were detected in tumors. The enhanced expression of miR-22, FXR and repressed CCNA2 level in the WA-treated tumors were observed by real-time PCR and Western blot analyses (Figure 6B). Immunohistochemical staning for Ki-67 demonstrated that the positive cells of tumors were significantly lower in WA-treated mice (Figure 6C). Collectively, WA inhibited tumor masses *in vivo* in a subcutaneous xenograft mouse model of HCC, the underlying insights of which were related to the miR-22-repressed CCNA2 pathway.

## DISCUSSION

**Figure 7:**
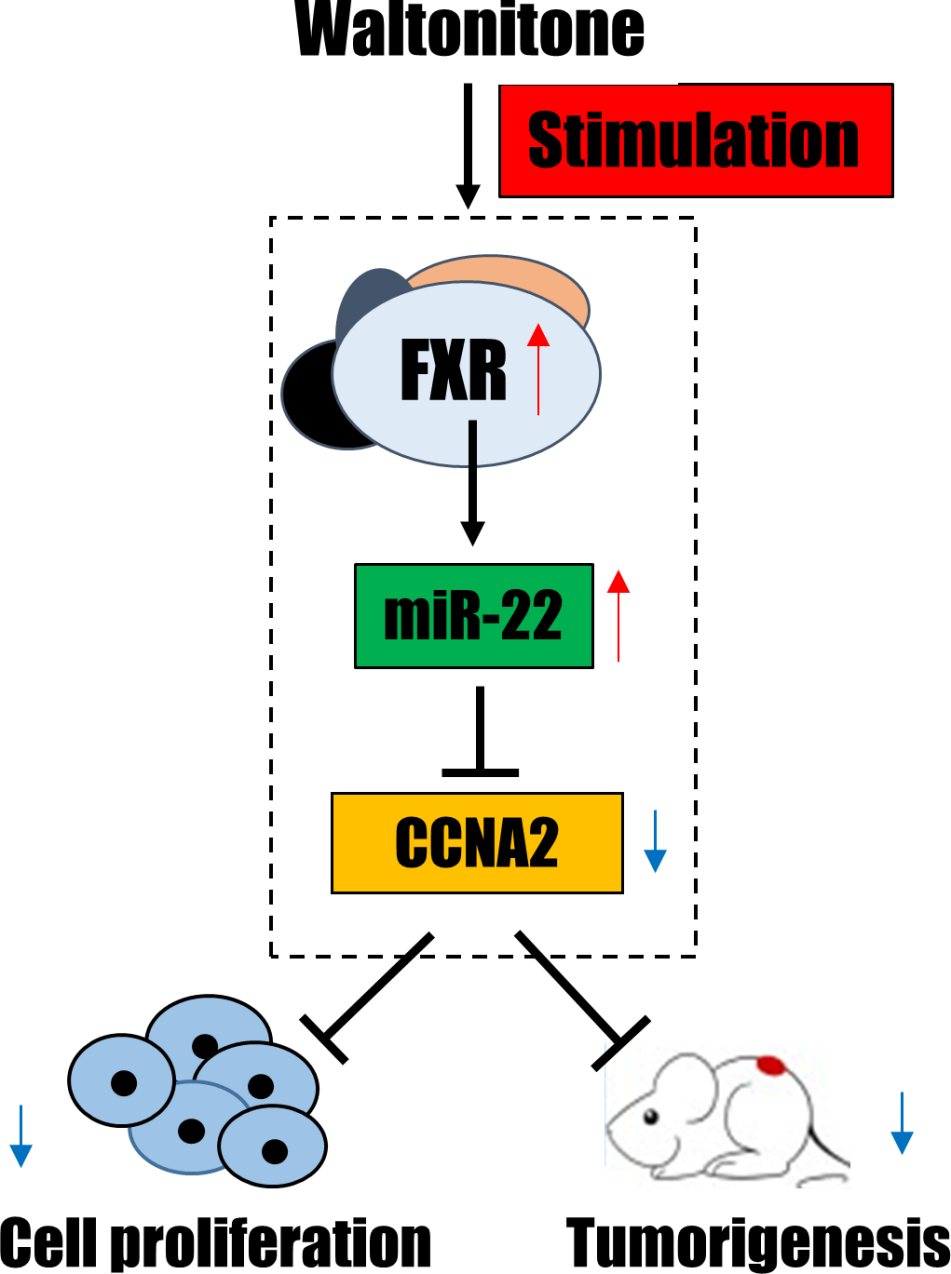
A summary diagram of the anti-tumor effect of WA through FXR-miR-22-CCNA2 axis

The present study revealed that WA induced miR-22 expression and repressed CCNA2, which was partially through FXR regulation. Moreover, evidence based on flow cytometry indicated that either miR-22 inhibition or FXR knockdown can partially reverse WA-induced HCC cell growth arrest. Furthermore, the sustained miR-22 overexpression along with repressed CCNA2 were observed after WA treatment *in vivo,* which was associated with decreased positive cell proliferation and tumorigenicity. Overall, the data showed that WA inhibited liver tumor progression through suppression of cell proliferation, in which the FXR-miR-22-CCNA2 axis was involved (Figure 7).

The diminished FXR expression has been largely reported in HCC tumorigenicity, and its low expression level was highly correlated with large tumor size, advanced stage and poor differentiation [18]. Previous report discovered that FXR silencing induced cell growth, migration, invasion in Huh7 cells and accelerated tumor xenografts formation in nude mice [19]. Our present data supported the findings that lower expression of FXR was observed in tumor tissues of HCC samples and knockdown of FXR abolished the anti-tumor effect of WA in *in vitro* study. Besides the role of FXR in HCC, repressed FXR expression was also observed in other cancer types, including cholangiocarcinoma [20], biliary tract carcinoma [21], and colon carcinoma [22]. However, the expression of FXR was not fully associated with tumorigenicity, overexpressed FXR in pancreatic cancer resulted in poor patient survivals, and inhibited FXR showed the beneficial effect of pancreatic tumor progression [23]. Other published documents argued that FXR expression was preserved and enhanced in human HCC as well. The data disclosed that increased or comparable intensity of nuclear FXR staining and lower expression of FXR was present in tumor tissues in comparison to non-tumorous tissues [24]. This controversial data maybe due to limitation of semi-quantitation or the discrepancy between individual samples. Importantly, the diminished FXR expression was an important event in tumor development in both mice and humans, and the best consideration for FXR as either a good marker to identify a high-risk subgroup of HCC patients or a potential therapeutic target for HCC treatment.

FXR-regulated multiple downstream signals were involved in liver carcinogenesis, and one of them was FXR-regulated miR-122 pathway. FXR positively induced miR-122 by directly binding to the DR2 element which are located in miR-122 promoter region, and FXR-regulated-miR-122 suppressed the proliferation of HCC cells and growth of HCC xenograft [25]. In the present study, we discovered another microRNA, miR-22 was transcriptionally regulated by FXR as well. Unlike the binding site as miR-122, FXR binds to the IR1 element in miR-22 promoter region, which has been proven by our previous study [17]. These observations indicate that microRNAs are the important targets of FXR and also discloses a potential approach to identify FXR-miRNAs interaction by FXR motifs searching. From our miRNA array data, we observed that the expression of miR-122 was upregulated by 2.5-fold when WA (25 μM) was treated for 48 h (data not shown), indicating miR-122 and miR-22 regulated downstream pathways were activated independently upon FXR stimulation. However, no clear evidence demonstrated which pathway contributed most to the tumor progression, thereby these two independent pathway may function in a cooperative fashion. Anyway, in the present study, we only focused on the FXR-miR-22-CCNA2 axis. Importantly, FXR mediated cell proliferation was highly involved in HCC formation. For instances, cyclin G1 was one of the target of miR-122 [25], cyclin D was also inhibited by FXR [19], and our study revealed the FXR-mediated cyclin A contributed to HCC cells proliferation. Together with these observations, FXR was a predominant regulator in HCC cells growth, and one major signal was related to cell cycle regulation.

Many reports on the mechanism of WA in treating HCC were well documented, most of which were focusing on cell apoptosis stimulation. WA induced cell apoptosis induction via the BCL family, which was identified as a potential drug candidate in liver carcinoma treatment. We believed there are other mechanisms contributed to the anti-tumor effect of WA in HCC treatment. The present study is the first to determine that WA can inhibit cell proliferation via the FXR/miR-22/CCNA2 pathway, which is responsible for HCC development. Different carcinoma cell lines may exhibit various responses to drugs. Thus, Huh7 and Hep3B, which are two commonly used liver cancer cell lines, were employed in this study. Both cell lines showed the same effect after WA treatment. The normal liver cell line, L02, was used for comparison in a specific cancer cell viability study after WA treatment. Our data indicated the inhibitory ability of WA exclusively affected on HCC cells but not on normal liver cells. Meantime, side effects, such as weight loss, hair loss, lethargy or dysphoria, macroscopically visceral pathogenic changes were not observed after WA treatment *in vivo* in current and even our previous studies [11]. All evidence indicated that WA could be developed as a safe drug for liver cancer treatment. However, the long-term exposure of WA in animals and comprehensive investigation of its toxicity are fully needed to confirm the safety of WA in clinical use.

MiR-22, which was regulated by FXR in our previous reports, served as a tumor suppressor in a wide range of human cancer types. Most of the miR-22-regulated targets involved in carcinoma development pathway were recently validated, including MYCBP [26], MCM7 [27], CDC25C [28], and CCNA2 [17], all of which are responsible for the cell proliferation. In our study, we focused the function of CCNA2, and the diminished CCNA2 after WA exposure was responsible for the HCC cell arrest in our study.

Additionally, we investigated the expressions of FXR, miR-22 and CCNA2 in tumor samples and non-tumorous tissues of HCC from available database and validated these three targets in another set of HCC. Collectively, the current clinical observation and our previous report suggested that targeting the FXR/miR-22/CCNA2 axis is a potential therapeutic option to develop drugs for liver cancer therapy.

Finally, to determine whether WA being a FXR agonist or activating FXR directly, LanthaScreen TR-FRET Farnesoid X Receptor Coactivator assay was conducted in the current study. Unfortunately, we failed to observe the dose-dependent activation of FXR upon WA treatment (data not shown). Nevertheless, the repression of CCNA2 by WA was eliminated in the absence of FXR. Thus, WA can inhibit HCC cell proliferation at least partially through FXR regulation. Indeed, additional studies are needed to verify whether WA is a FXR agonist. Besides that, *in vitro* study, when we overexpressed FXR in cells and subsequently treated with WA, we found that the protein level of CCNA2 was promisingly inhibited when comparing with WA treatment alone (Supplementary Figure S4), which indicating the synergistic effect of WA and FXR agonist, but further investigation are still warranted. Together, the presented evidence suggested that WA can inhibit HCC cell proliferationand tumorigenesis through miR-22-repressed CCNA2, which was at least partially through FXR regulation (Figure 7).

## MATERIALS AND METHODS

### Materials

Waltonitone was obtained from Shanghai R&D Centre for Standardization of Chinese Medicines (No.07–2013, purity 98%). The structure of waltonitone was reported by our previous study [11]. The miR-22 inhibitor and siRNAs of FXR were obtained from GenePharma (GenePharma, Shanghai, China) and the sequences were listed in Supplementary Table S1. The reagents used for cell culture were purchased from Gibco-BRL (Carlsbad, CA, USA) unless otherwise specified.

### Cell culture

Huh7 (Japanese Collection of Research Bioresources), Hep3B (American Type Culture Collection), and L02 (Cell Bank of Type Culture Collection of the Chinese Academy of Sciences) were cultured in Dulbecco's modified Eagle's medium supplemented with 10% fetal bovine serum. Cells were plated (1.5 × 10^6^ cells per 60 mm dish, 3 × 10^5^ cells per 6-well plates, and 3 × 10^4^ cells per 24-well plates) overnight prior to treatment or transfection. Cells were serum-starved for 24 h prior to waltonitone treatment, and cells were subjected to waltonitone exposure in serum-free media as well except for the colony formation experiment, which was employed in the media within 1% FBS.

### Cell viability assay

Cells were plated in 24-well plates overnight prior to WA treatment. The doses of WA at 5, 10, 25, and 50 μM were used for 12, 24, and 48 h of treatment. Up to 50 μl of CCK-8 solution was added to each well and incubated for 1 h to 4 h (Shanghai Yeasen Biotechnology, Shanghai, China). Finally, the absorbance of the sample at 450 nm was measured using a microplate reader (Thermo Scientific, USA). The experiments were repeated three times under same condition.

### Colony formation assay

A total of 2500 cells per well were plated in 12-well plates. Cells were treated for 15 days with WA at a concentration of 5, 10 and 25 μM before clear colonies were observed (within 1% FBS). Cells were gently washed with PBS, fixed with 3.7% formaldehyde for 10 min, and stained with 0.2% crystal violet solution in 10% ethanol for 10 min. PBS was utilized to wash off the excess crystal violet. The experiments were repeated three times under same condition.

### Cell-cycle assay

The experiments were conducted by transfection with either miR-22 inhibitor or siFXR for 6 h, and followed by WA (25 μM) or DMSO treatment for 48 h. For each treatment group, 2 × 10^6^ cells were collected in PBS and fixed overnight in 70% ethanol at −20°C. Cells were resuspended in 300 μl of propidium iodide staining buffer and incubated for 30 min at room temperature. DNA content analyses were performed using FACScan flow cytometry (Becton Dickinson, CA, USA). Each individual experiment was repeated three times under same condition.

### Quantification of RNA

Total RNA was extracted using TRIzol reagent (Invitrogen, Carlsbad, CA) and reverse transcribed into cDNA by using Primescript Reverse Transcription Master Mix (TaKaRa, Japan). miR-22 and mRNA expression levels were quantified by Real-time PCR on VII7 system (Applied Biosystems, CA) by employing SYBR-Green PCR Master Mix (Applied Biosystems, CA). The primers were designed using Primer3 Input online version, and the primer sequences are listed in Supplementary Table S2. U6 and GAPDH were utilized as internal controls to normalize the miR-22 and mRNA levels. The RNA were obtained from three individual experiments under same condition.

### Western blot

The experiments were conducted by transfection with either miR-22 inhibitor or siFXR for 6 h, and followed by WA (25 μM) or DMSO treatment for 48 h. Cells were lysed with RIPA protein extraction reagent (Thermo Scientific, Rockford, IL, USA). Proteins (50 μg) were electrophoresed by 10% SDS-PAGE and transferred onto a PVDF membrane (Bio-Rad, Hercules, CA). Nonspecific binding was blocked with 5% nonfat milk in TBST (10 mM Tris pH 7.5, 100 mM NaCl, 0.1% Tween 20) for 2 h at room temperature and incubated overnight at 4°C with an anti-CCNA2 antibody (Abcam, Cambridge, MA). Goat anti-mouse IgG (Santa Cruz Biotechnology, Santa Cruz, CA) was used to detect CCNA2. Signals were detected using ECL system SuperSignal West Pico Chemiluminescent Substrates (Pierce, Rockford, IL). Protein levels were normalized to β-actin levels (Santa Cruz Biotechnology, Santa Cruz, CA). The proteins were obtained from three individual experiments under same condition.

### Immunofluorescence

Cells were seeded onto 0.8 cm × 0.8 cm coverslips overnight in a 24-well plate prior to WA treatment (25 μM). At 48 h after treatment, cells were fixed with 4% paraformaldehyde for 15 min at room temperature. After three rinses in PBS, cells were blocked with 3% bovine serum albumin and 0.03% tritonx-100 for 90 min at room temperature and subsequently incubated with primary antibody (anti-CCNA2, 1:100) overnight (Abcam, CA, USA). After rinsing with PBS, cells were incubated with conjugated secondary FITC-labeled antibodies for 1 h (Abcam, CA, USA). After three times washing by PBS, coverslip slides were added Prolong Gold Anti-Fade Reagent with DAPI (CST) and observed under a fluorescence microscope in at least six microscope fields for each section (Olympus, Japan).

### Ki-67 immunohistochemical staining

Ki-67 immunohistochemical staining was performed with primary Ki-67 antibody (Abcam, CA, USA) to monitor cell proliferation in tumors of WA-treated or untreated mice. The number of Ki-67-labeled nuclei was determined by counting the Ki-67-positive cells in at least six microscope fields for each section, and the experiments were repeated three times for statistics analysis (Olympus, Japan).

### *In vivo* study

Male BALB/c nude mice were purchased from the Laboratory Animal Center of Shanghai University of Traditional Chinese Medicine (SHUTCM, Shanghai) and housed at 20 ± 2°C with a relative humidity at 60% to 70% under specified-pathogens free level. The animal welfare was strictly complied with the Guide for the Care and Use of Laboratory Animals, and the protocols for the animals experiment were approved by the Institutional Animal Committee of Shanghai University of Traditional Chinese Medicine (Permit number: SYXK (Hu) 2014–2008). Huh-7 cells (2 × 10^6^/ml) in PBS were subcutaneously injected into the right flank of mice. Eight to ten mice were included in each group. One week later after successful tumor transplantation, WA or vehicle was intraperitoneally (i.p.) injected once every 2 days for the succeeding 15 days. The dose of WA at 50 mg/kg was used in this study. At the end of the treatment, all mice were sacrificed, and tumors were removed. The tumors were immediately transferred and stored carefully for further Real-time PCR and western blot assay, which were demonstrated above. In the whole treatment, body weights of each mouse were monitored every three days, and tumor size was measured in day 3, day 8 and day 15.

### Clinical data

The miRNA and mRNA expression data of 80 paired clinical HCC samples were downloaded from GEO database (GSE22058). The downloaded series matrix files contain miRNA and mRNA data, and the relative expression levels of FXR, miR-22 and CCNA2 were selected for correlation analysis. Another set of HCC samples (12 human hepatocellular carcinoma and 9 normal liver specimens) was used for the expression pattern validation. Among them, 6 tumors and adjacent normal tissues were paired and derived from 6 patients. Anonymized liver tissues were obtained according to guidelines approved by the Institutional Review Board of the University of California, Los Angeles., which have been described in our previous study [17]. According to UCLA policy, investigators who use completely anonymized human tissue samples do not need IRB approval. Details regarding this policy can be found on the UCLA Translational Pathology Core Laboratory web site (http://www.pathology.medsch.ucla.edu/tpcl/pages/feesOrdering).

### Data analysis

One-way ANOVA and *T*-test statistics were performed using SPSS 20.0 (SPSS Inc. Chicago, IL, USA). Plots were generated by GraphPad Prism 5.0 (GraphPad Software, Inc., San Diego, CA) or SPSS (SPSS Inc. Chicago, IL, USA).

## SUPPLEMENTARY MATERIALS FIGURES AND TABLES


