# Supplementary material for: Waltonitone inhibits proliferation of hepatoma cells and tumorigenesis via FXR-miR-22-CCNA2 signaling pathway
Source: Oncotarget. 2016 Oct 12;7(46):75165–75. doi: 10.18632/oncotarget.12614 (PMC5342731; doi:10.18632/oncotarget.12614)
Supplement: Supplementary file 1 [file oncotarget-07-75165-s001.pdf]

Waltonitone inhibits proliferation of hepatoma cells and tumorigenesis via FXR-miR-22-CCNA2 signaling pathway

Supplementary Materials

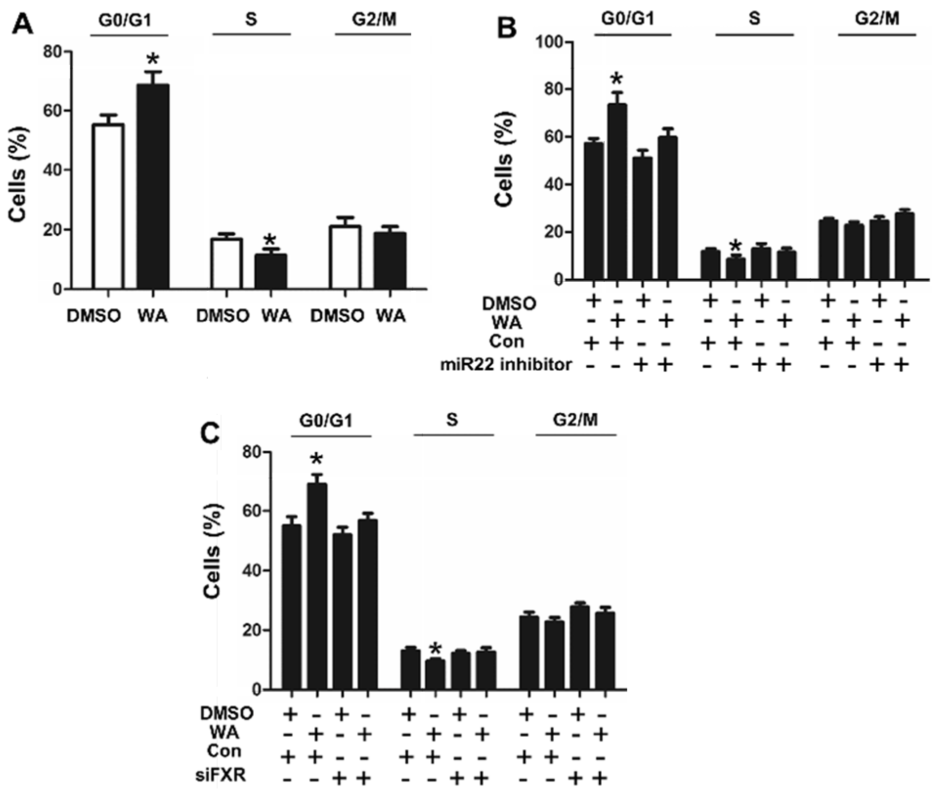

Supplementary Figure S1: Visualized box plots of cell cycle analysis. After transfection with miR-22 inhibitor or siFXR for 6 h, WA (25 μM) or DMSO were treated for 48 h. Cell cycle analysis was conducted by using flow cytometer (**A–C**). The experiments were repeated three times. $^{*}p < 0.05$, versus DMSO+Con.

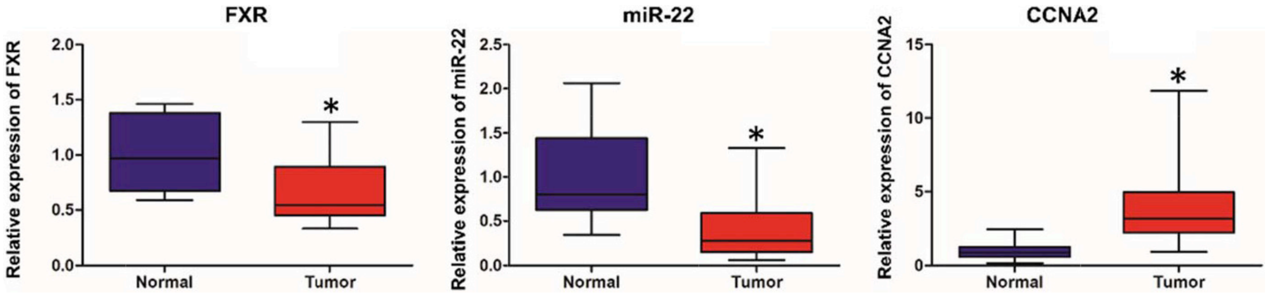

Supplementary Figure S2: The expression level of FXR, miR-22 and CCNA2 in HCC samples and normal ones. Twelve human hepatocellular carcinomas (HCC) and nine normal liver specimens were included in the study [17]. Among them, 6 tumors and adjacent normal tissues were paired and derived from six patients. $^{*}p < 0.05$.

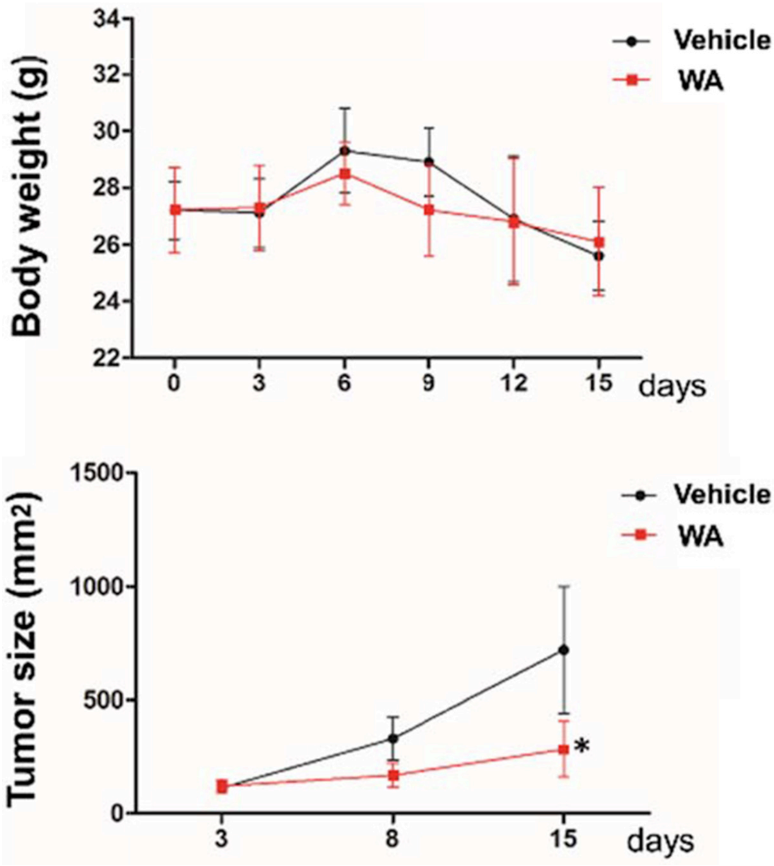

Supplementary Figure S3: The body weights and tumor size of WA-treated or untreated mice. Eight mice were included in each group. The body weights of mice were monitored every three days, and each tumor size was measured in day 3, day 8 and day 15. $^{*}p < 0.05$, versus Vehicle.

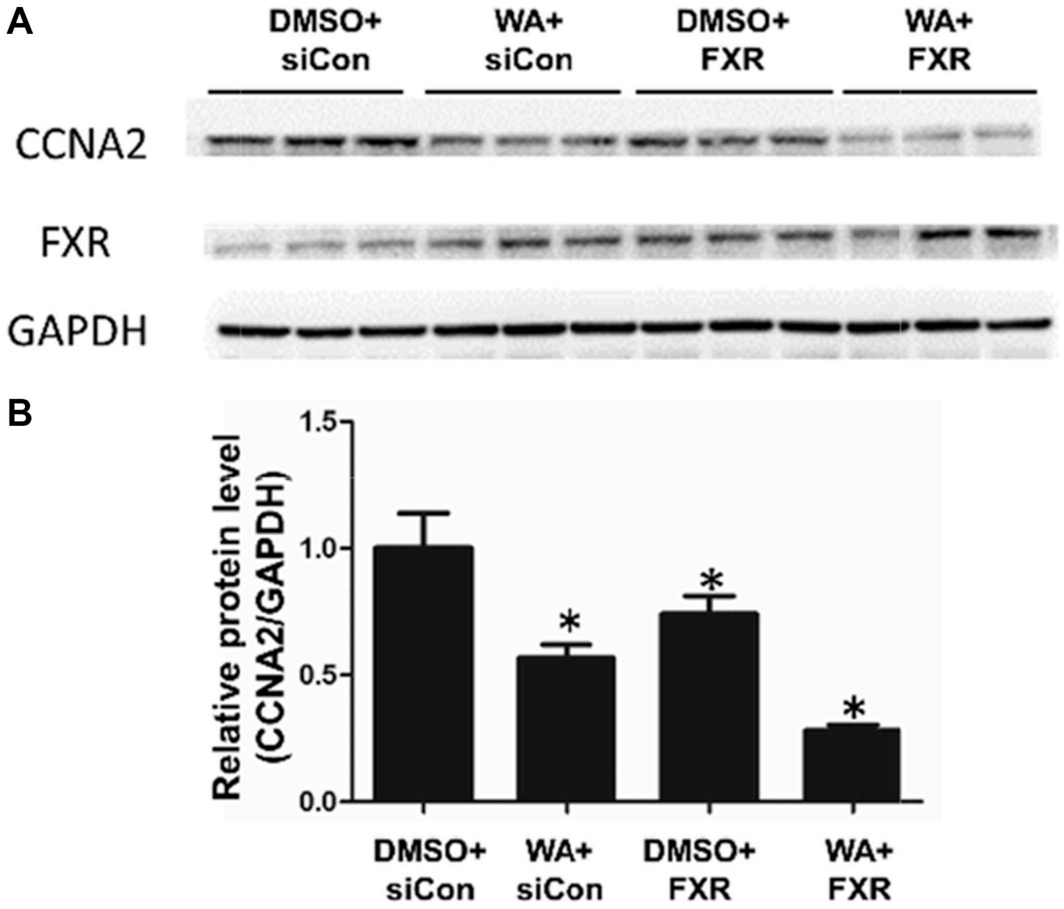

Supplementary Figure S4: Protein level of CCNA2 was measured upon overexpressed FXR and WA treatment. Overexpressed FXR and RXRα plasmids (total, 500 ng) were co-transfected in Huh-7 cells prior to WA treatment (25 μM for 48 h). The experiments were repeated three times. $^{*}p < 0.05$, versus DMSO+siCon.

Supplementary Table S1: The sequences of siRNAs were used in our study

Negative sequence	5′-TTCTCCGAACGTGTCACGTTT-3′
siFXR1	5′-GCGGTTGAAGCTATGTTCCTTCGTT-3′
siFXR2	5′-GGCTCCAGGGAATCCTGCATTCTAA-3′
siFXR3	5′-GCTGGATCCCGTCTGGGCATTCTGAC-3′
siFXR4	5′-CGCACTCGAGTCACTGCACGTCCCAGATT-3′
miR-22 inhibitor	5′-ACAGUUCUUCAACUGGCAGCUU-3′

Supplementary Table S2: The sequences of primers were used in our study

	Forward (5′-3′)	Reverse (5′-3′)	Stem-loop (5′-3′)
FXR (human)	TGGGGAACTG AAAATGACTC	ACAGGCAAAGT GTTGAGGAT	
CCNA2 (human)	CCTGCAAACT GCAAAGTTGA	AAAGGCAGCTCC AGCAATAA	
miR22 (human)	CGCGAAGCTGCC AGTTGAAG	GTGCAGGGT CCGAGGT	GTCGTATCCAGTGCAGGGTCCGAGGTA TTCGCACTGGATACGACACAGTT
FXR (mouse)	TTCCTCAAGTT CAGCCACAG	TCGCCTGAGTTC ATAGATGC	
CCNA2 (mouse)	TGAGAATGG AGCACCTAG	TCTGTTGTGC CAATGACT	
miR22 (mouse)	CGCGAAGCTGC CAGTTGAAG	GTGCAGGGT CCGAGGT	GTCGTATCCAGTGCAGGGTCCGAGGTATT CGCACTGGATACGACACAGTT
